# *Schistosoma* Egg Antigen Induces Oncogenic Alterations in Human Prostate Cells

**DOI:** 10.1155/2018/4675380

**Published:** 2018-12-09

**Authors:** Isaac Tuffour, Irene Ayi, Theresa Manful Gwira, Edward Dumashie, Yvonne Ashong, Regina Appiah-Opong

**Affiliations:** ^1^West African Centre for Cell Biology of Infectious Pathogens, Department of Biochemistry, Cell and Molecular Biology, College of Basic and Applied Sciences, University of Ghana, P.O. Box LG 54, Legon, Accra, Ghana; ^2^Department of Biochemistry, Cell and Molecular Biology, College of Basic and Applied Sciences, University of Ghana, P.O. Box LG 54, Legon, Accra, Ghana; ^3^Department of Clinical Pathology, Noguchi Memorial Institute for Medical Research, College of Health Sciences, University of Ghana, P.O. Box LG 581, Legon, Ghana; ^4^Department of Parasitology, Noguchi Memorial Institute for Medical Research, College of Health Sciences, University of Ghana, P.O. Box LG 581, Legon, Ghana

## Abstract

Schistosomiasis is a neglected tropical disease that affects 200 million people and accounts for 100,000 deaths annually. In endemic geographical areas, schistosomiasis has been implicated as an etiological agent in the pathogenesis of bladder, colorectal, and renal carcinoma largely due to *Schistosoma* eggs in tissues that comes with chronic infection. Several studies have also reported cases of association between *Schistosoma* infection and prostate cancer. The possible causal association is however poorly understood. We hypothesized in this study that infection of the prostate cells with *Schistosoma spp* promotes cancer. Urine samples from individuals living in Galilea, a schistosomiasis endemic community in the Ga South District of Ghana, were collected and screened for *Schistosoma* infection via microscopy and multiplex PCR. Soluble egg antigens (SEA) were prepared from *Schistosoma* egg-positive urine samples and assessed for the ability to induce cancer-like phenotypes including excessive proliferation, oxidative stress (reduced glutathione (GSH) depletion), and diminished apoptosis in cultured human prostate (PNT2) cells. Molecular analysis revealed infecting schistosome species to be *S*. *haematobium* and *S. mansoni*. Prostate cell proliferation was significantly induced by 12.5 *μ*g/ml SEA (*p* = 0.029). Also, SEA dose-dependently depleted cellular GSH. Flow cytometric analysis and fluorescence staining revealed that SEA dose-dependently diminished apoptosis, significantly, in prostate cells. Findings of this study suggest that schistosome infection may play a role in the pathogenesis of prostate cancer. *In vivo* studies are however needed to confirm this association.

## 1. Introduction

Prostate cancer is an important global health challenge. It represents the highest form of cancer and the commonest cause of cancer death in men from the United States of America and Northern Europe [1]. About 99% of prostate cancer cases occur in men above the age of 50 years, and the disease is characterized by painful urination, blood in urine, frequent urination, and sexual function disorders including difficulty in achieving erection and painful ejaculation [2]. Despite the overwhelming escalation of the disease and its burden globally, much is not known about its etiology. However, factors such as old age, race, genetic, and environmental factors are suspected to increase the risk of prostate cancer [3].

The role of infectious diseases in the etiology of prostate cancer is largely unknown. Many studies have, however, reported cases of association between the disease and schistosomiasis. Earlier studies reported the presence of *Schistosoma* eggs in 20% of 200 cadavers and 50% of prostate and seminal vesicles, respectively, in regions with high schistosomiasis prevalence [4, 5]. Similarly, several clinical cases have been reported on the presence of schistosome eggs in prostate biopsies and surgery-obtained tissues from prostate cancer patients in various schistosomiasis endemic geographical areas [6–8]. The average age of most of these schistosomiasis-associated prostate cancer patients seems relatively lower than the age category (≥50 years) normally ascribed to individuals with prostate cancer. For instance, Cohen and colleagues reported on advanced prostate cancer associated with multiple S*. haematobium* eggs in three young adults (one aged 27 and two 29 years) from South Africa [9]. Interestingly, none of these patients were confirmed to have any family history of prostate cancer. This and many other related case reports suggest that infection with *Schistosoma* parasite and deposition of the *Schistosoma* eggs in prostate tissues may contribute to the pathogenesis or progression of prostate cancer.

*Schistosoma haematobium* infection has been classified as a group 1 biocarcinogen by the International Agency for Research on Cancer (IARC)—WHO. However, the cellular and molecular mechanisms linking infection with *S. haematobium* and carcinogenesis are yet to be defined. It has been known for several decades that squamous cell carcinoma of the bladder cancer was geographically associated with urogenital schistosomiasis in regions with high risk of exposure to *S. haematobium* infection [10, 11]. Schistosome worm and egg-derived estrogen-like molecules and their metabolites have been postulated as the key carcinogenic substances implicated in schistosomiasis-linked cancers. A study conducted in 2015 on urine and serum samples of 40 Angolan men who were concomitantly infected with *S*. *haematobium* and diagnosed with bladder cancer discovered the presence of unique estrogen-like metabolites which were not reported in the urine metabolome of healthy humans [12]. Among these metabolites were catechol estrogen quinones (CEQs) and their DNA adducts. These estrogen metabolites have been speculated to react covalently with DNA bases by forming depurinated sites. Error-prone repair of the modified DNA has been reported to generate oncogenic alterations which are evidenced in increased cell proliferation, upregulation of oncogenes, down-regulation of tumor suppressor genes, and diminished apoptosis [13].

There has not yet been any report on empirical data proving the relationship between schistosome infection and prostate cancer. This present study therefore sought to ascertain the oncogenic potential of *Schistosoma spp* soluble egg antigen (schSEA) in human prostate cell using *in vitro* cellular and biochemical approaches.

## 2. Materials and Methods

### 2.1. Urine Sample Collection and Identification of *Schistosoma spp* Eggs

The study was conducted in Galilea, a schistosomiasis endemic community along the Densu Lake in the Ga South District of the Greater Accra Region of Ghana in 2017. Urine was obtained from 210 individuals of both genders following ethical approval and informed consent from community leaders and participants. Urine samples were obtained following interaction with participants for presumptive diagnosis and instruction for collection. *Schistosoma* infection was detected by microscopic observation of eggs morphologically in the sediment of centrifuged urine [14]. Confirmation of infection and identification of infecting species was done by detecting the presence of schistosome DNA in all the urine samples by PCR using species-specific primers. All urine samples were kept at 4°C for harvesting of eggs after PCR.

Ethical approval for the study was obtained from the NMIMR Institutional Review Board (NMIMR-IRB CPN 069/16-17).

#### 2.1.1. DNA Isolation

DNA was extracted from urine samples using QIAamp DNA Mini Kit (Qiagen, Hilden, Germany) according to the manufacturer's instructions. The extracted DNA was quantified using NanoDrop ND-1000 spectrophotometer (NanoDrop Technologies, USA) and stored at −20°C until use.

#### 2.1.2. PCR Amplification

*Schistosoma* species-specific primers amplifying the schistosome partial COX1 mitochondrial DNA region, consisting of a universal forward primer ShbmF (5′-TTTTTTGGTCATCCTGAGGTGTAT-3′) with three species-specific reverse primers, ShR (5′-TGATAATCAATGACCCTGCAATAA-3′) for *S. haeamtobium*, SbR (5′-CACAGGATCAGACAAACGAGTACC-3′) for *S. bovis*, and SmR (5′-TGCAGATAAAGCCACCCCTGTG-3′) for *S. mansoni*, were used for amplification of extracted DNA [15].

PCR amplification was performed in a reaction mixture containing master mix (Qiagen Multiplex PCR HotStarTaq DNA Polymerase, Hilden, Germany), 1.6 *μ*M of the universal forward primer (ShbmF), 0.8 *μ*M of each of the three reverse primers (ShR, SbR, and SmR), and DNA (~103.7 ng/*μ*l from urine samples). PCR cycling conditions consisted of an initial denaturing step of 95°C for 15 min, followed by 30 cycles of 94°C for 30 s, 58°C for 1 min and 30 s, and 72°C for 1 min and 30 s, with a final extension time of 10 min at 72°C. Amplicons were visualized on a 2% agarose gel for the expected band sizes (375 bp for *S. mansoni*, 543 bp for *S. haematobium*, and 306 bp for *S. bovis*).

### 2.2. Preparation of *Schistosoma spp* Soluble Egg Antigens

Urine samples of *S. haematobium*- and/or *S. mansoni*-infected persons were centrifuged at 1500 rpm for 5 min, and supernatants were discarded. Pellets (containing schistosome eggs) were pooled and suspended in phosphate-buffered saline (PBS) and washed 3 times by centrifuging at low speed. Resulting pellet of eggs was then frozen at −80°C for 10 min. The frozen pellet was ultrasonified for 2 min in a repeated cycle of 5 times. When approximately 95% of the eggs were disrupted, the lysate was centrifuged for 20 min at 1000 rpm at 4°C (Hitachi CF7D2, Japan). The supernatant was then collected and ultracentrifuged for 90 min at 14,000 rpm at 4°C. The supernatant which was the *Schistosoma spp* soluble egg antigen (schSEA) was filter-sterilized and stored at −80°C until use [14]. Protein concentration of SEA was determined by the Bradford method [16].

### 2.3. Determination of Effect of schSEA on Human Normal Cells

#### 2.3.1. Cell Culture

Normal human prostate (PNT2) cells obtained from RIKEN BioResource Centre Cell Bank (Japan) were maintained in RPMI-1640 culture medium enriched with 10% fetal bovine serum (FBS) and 1% penicillin-streptomycin and incubated at 37°C in the presence of 5% CO_2._ The cells were subcultured every week until ready for use. Prior to treatment with schSEA for determination of effects, the cells were serum-starved overnight. Appropriate assays were run on schSEA-treated cells to determine induction of oxidative stress, effect on cell proliferation, apoptosis, and integrity of nuclei.

#### 2.3.2. Oxidative Stress Assay

Oxidative stress-inducing effect of schSEA was determined by evaluating the levels of cellular reduced glutathione (GSH) [17]. Following exposure to schSEA, cells (2 × 10^5^ cells/ml) were detached from 24-well plates, harvested, and homogenized by ultrasound with 5% trichloroacetic acid for 15 min. The cell homogenates were centrifuged for 30 min at 3000 rpm (Hettich zentrifugen MIKRO 200, Germany), and supernatants were treated with 10 mg/ml o-phthalaldehyde (OPA). Following incubation in the dark for 15 min, GSH levels in the supernatant were obtained by measuring at wavelength of 340 nm (excitation) and 460 nm (emission).

#### 2.3.3. Cell Proliferation Assay

The proliferative effect of schSEA was determined using the CellTiter 96 AQ nonradioactive cell proliferation assay [18]. Briefly, cells were plated at 1 × 10^4^ cells/well in a 96-well plate and challenged with varying concentrations (0-50 *μ*g/ml) of the schSEA for 24 h and incubated at 37°C. The 3-(4,5-dimethylthiazol-2-yl)-5-(3-carboxymethoxyphenyl)-2-(4-sulfophenyl)-2H-tetrazolium (MTS) solution was then added to each well, and cells were further incubated for 4 h at 37°C. The amount of formazan produced (to indicate a measure of cell viability) was measured at a wavelength of 490 nm with a microplate reader (Tecan Infinite PRO, Austria). Absorbance values were recorded and used to evaluate the percentage increase in proliferation for each treatment concentration relative to the negative control by using the following formula:

(1) $$\begin{array}{c} \%cell\;proliferation=\left[\frac{\left(absorbance\text{ of }schSEA\;treatment-absorbance\text{ of }negative\;control\right)}{absorbance\text{ of }negative}\right]*100\%. \end{array}$$

#### 2.3.4. Apoptosis Assay

The effect of schSEA on apoptosis was elucidated by flow cytometry. Briefly, cells (2 × 10^5^ cells/ml) were cultured overnight in 24-well plates and challenged with varying concentration (0-12.5 *μ*g/ml) of schSEA for 24 h. The cells were then harvested and treated with equal volumes of guava nexin reagent (contains annexin V-FITC and 7-AAD), incubated for 20 min in the dark at room temperature, and analyzed immediately after incubation with a flow cytometer (Guava EasyCyte HT).

#### 2.3.5. Nuclei Integrity Analysis

Effect of schSEA on cell nuclei integrity was determined using the cell permeable DNA dye Hoechst 33258 [19]. Briefly, cells were cultured overnight in 24-well plates at a density of 2 × 10^5^ cells/ml and treated with varying concentration (0-12.5 *μ*g/ml) of schSEA for 24 h. The cells were collected from individual wells, washed once with PBS, and fixed with 1% glutaraldehyde. After 30 min incubation at room temperature, cells were centrifuged and supernatants were discarded. Cell pellets were then stained with 1 mM Hoechst 33258. Stained cells were observed under a fluorescence microscope equipped with a digital camera to determine the extent of nuclei condensation.

### 2.4. Statistical Analysis

Microsoft Excel 2013 and the GraphPad Prism version 6 were used to analyze the results. Data were expressed as mean ± SD. Student's *t*-test was used to assess statistical significance of differences between test (schSEA-treated cells) and controls (serum-fed cells and shSEA-untreated cells). *p* ≤ 0.05 was considered statistically significant.

## 3. Results

### 3.1. Prevalence of *S. haematobium* and *S. mansoni* Infection

**Figure 1:**
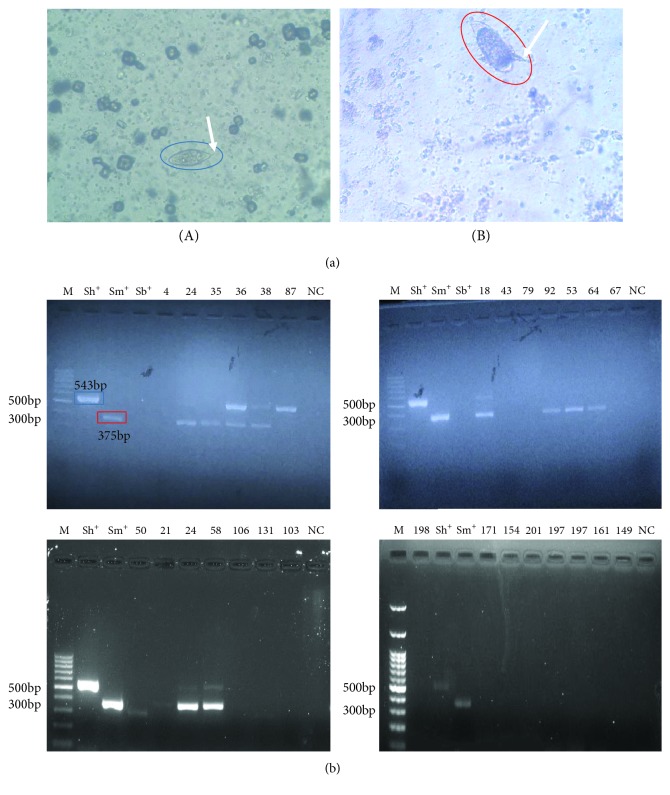
(a) *Schistosoma* eggs in urine of infected participants: (A) *S. haematobium* egg (terminal spine indicated with arrow) and (B) *S. mansoni* egg (lateral spine indicated with arrow). Eggs were visualized under an Olympus CX12 compound microscope (magnification ×20). (b) Amplification of the partial COX1 gene of *Schistosoma* spp. Lane M = 100 bp molecular weight marker; lane Sh^+^ = *S*. *haematobium* DNA (positive) control; lane Sm^+^ = *S. mansoni* DNA (positive) control; lane Sb^+^ = *S. bovis* DNA (positive) control; lane NC = negative control (nuclease-free water). Numbered lanes are DNA from participants' urine samples.

Microscopically, schistosome eggs were found in 30 (14.4%) of the urine samples. Both *S. haematobium* and *S. mansoni* eggs were found in 11 urine samples; 10 of them were from male participants and 1 from a female participant. The remaining 19 urine samples (11 males and 8 females) were detected to be infected with S. *haematobium* eggs only (Figure 1(a)).

However, 21 (10.0%) participants were found to be positive for schistosome infection by PCR. Out of 21 participants, 7 (33.3%) were found with *S. haematobium* infection only, 9 (42.9%) with *S. mansoni* infection only, and 5 (23.8%) with *S. haematobium* and *S. mansoni* coinfection. None of the samples were found positive for *S. bovis* (Figure 1(b)).

### 3.2. *Schistosoma spp* SEA Induces Oxidative Stress in PNT2 Prostate Cells

**Figure 2:**
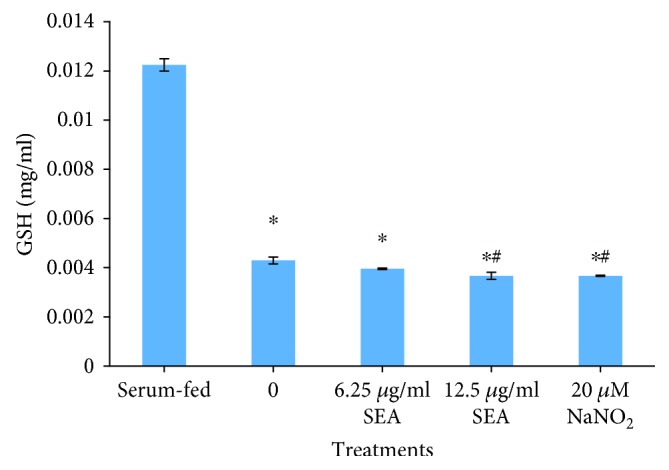
schSEA depletes GSH levels in prostate (PNT2) cells. Statistical significance by Student's *t*-test; ^∗^*p* ≤ 0.05 (serum-fed vs. serum-starved experiments) and ^#^*p* ≤ 0.05 (non-schSEA treatment vs. schSEA-treated cells/NaNO_2_-treated cells). Results are representative of three independent experiments.

Reduced glutathione level is used to assess the oxidative status of a cell. Following treatment of cells, it was observed that schSEA dose-dependently and significantly depleted cellular GSH levels in treated cells (*p* = 0.001) as compared to serum-fed cells. Additionally, 12.5 *μ*g/ml SEA significantly depleted GSH levels in comparison to non-schSEA-treated serum-starved cells (negative control) (*p* = 0.023). Sodium nitrite- (NaNO_2_-) treated serum-starved cells were included as positive control (Figure 2).

### 3.3. *Schistosoma spp* SEA Induces Proliferation of PNT2 Prostate Cells

**Figure 3:**
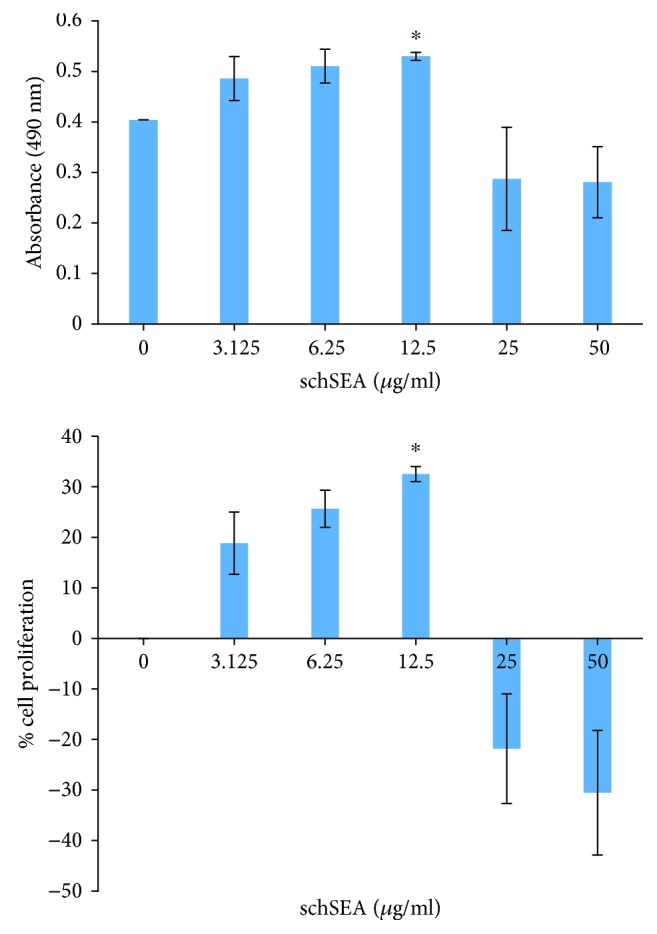
schSEA induces proliferation of prostate (PNT2). Statistical significance by Student's *t*-test: ^∗^*p* ≤ 0.05 (non-schSEA treatment vs. 12.5 *μ*g/ml schSEA-treated cells). Results are representative of three independent experiments.

The SEA dose-dependently increased cell viability and proliferation up to the concentration of 12.5 *μ*g/ml (*p* = 0.03). Subsequently, there was a drastic decline of proliferation at schSEA concentrations of 25 *μ*g/ml and 50 *μ*g/ml (Table 1 and Figure 3).

### 3.4. *Schistosoma spp* SEA Decreases Apoptosis of PNT2 Prostate Cell

**Figure 4:**
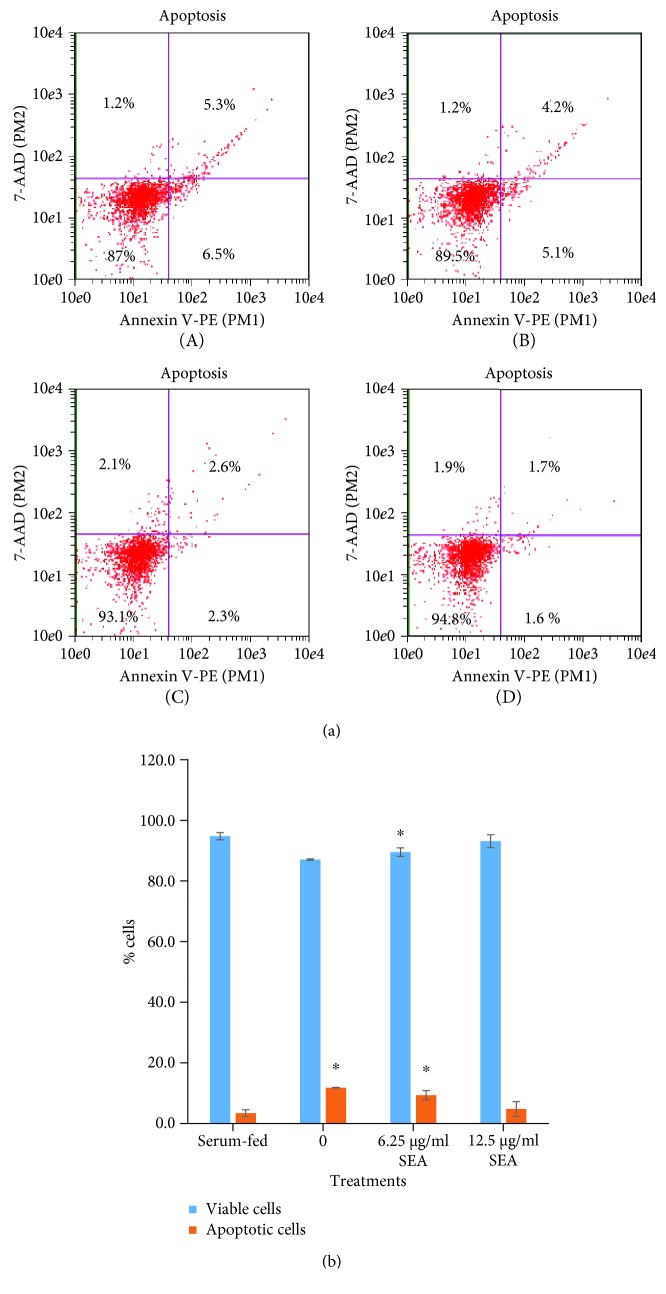
schSEA diminishes apoptosis in PNT2 cells. (a) (A) Negative control, (B) 6.25 *μ*g/ml schSEA, (C) 12.5 *μ*g/ml schSEA, and (D) serum-fed (positive control). Lower left quadrant: viable cells; lower right quadrant: early apoptotic cells; upper left quadrant: nuclear debris; upper right quadrant: late apoptotic cells. Data was acquired by Guava EasyCyte™ flow cytometer. (b) Comparison of apoptosis and cell viability among various experimental groups. Statistical significance by Student's *t*-test: ^∗^*p* ≤ 0.05 (serum-fed vs. serum-starved/schSEA-treated groups). Results are representative of three independent experiments.

The schSEA-treated cells recorded a concentration-dependent decrease in percentage of apoptotic cells relative to the untreated cells/negative control (i.e., 11.8%, 9.3%, and 4.9% for the negative control, 6.25 *μ*g/ml schSEA, and 12.5 *μ*g/ml schSEA-treated cells, respectively). Serum-fed cells (positive control) recorded the lowest percentage (3.3%) of apoptotic cells (Figure 4(a)). Conversely, SEA-treated cells recorded a concentration-dependent increase in the percentage of viable cells. The increase in percentage of viable cells followed the order: serum-fed cells (94.8%) > 12.5 *μ*g/ml SEA-treated cell (93.1%) > 6.25 *μ*g/ml SEA-treated (89.5%) > untreated cells (87%). There was significant difference in the percentage of viable cells between the serum-fed cells and untreated cells (*p* = 0.03) and 6.25 *μ*g/ml SEA-treated cells (*p* = 0.02). However, no significant difference was found between serum-fed cells and 12.5 *μ*g/ml SEA-treated cells. There was a significant difference in the percentage of apoptotic cells between the serum-fed cells and untreated cells (*p* = 0.03) and SEA-treated cells (*p* = 0.02) (Figure 4(b)).

### 3.5. *Schistosoma spp* SEA Decreases Nuclei Condensation of PNT2 Prostate Cells

**Figure 5:**
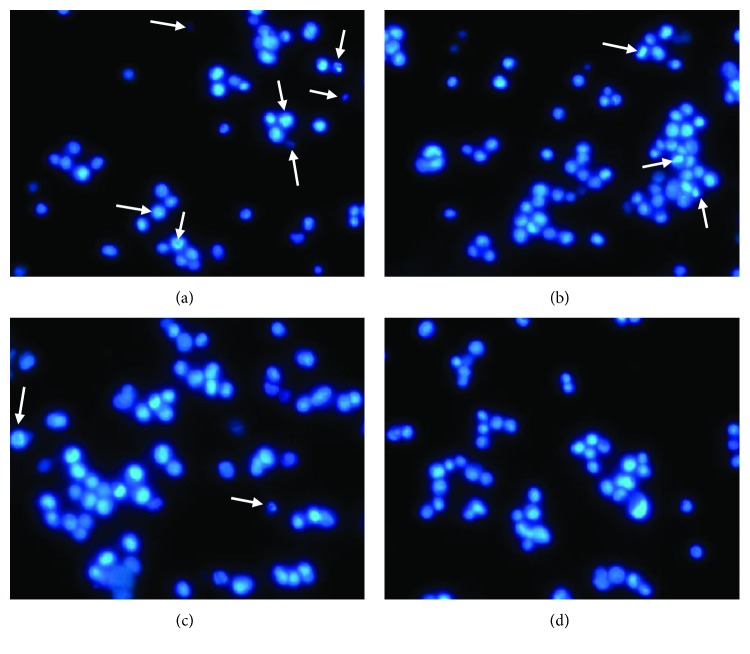
schSEA diminishes nuclei condensation in PNT2 cells: (a) negative control, (b) 6.25 *μ*g/ml schSEA, (c) 12.5 *μ*g/ml schSEA, and (d) serum-fed (positive control). Condensed and morphologically altered nuclei indicated by arrows. Cells were stained with Hoechst 33258 dye and nuclei visualized with fluorescence microscope (magnification 40x) (Olympus DP 72, Japan).

As shown in Figure 5, the untreated cells displayed most nuclei morphological changes characterized by chromatin condensation/nuclei shrinkage. The SEA-treated cells exhibited dose-dependent reduction in the number of morphologically condensed nuclei. The serum-fed cells on the other hand presented intact nuclear morphology.

## 4. Discussion

Schistosomiasis, one of the major neglected tropical diseases, has been implicated in several case report studies as a possible etiological agent in prostate cancer pathogenesis. In this study, we elucidated the oncogenic effect of *Schistosoma spp* soluble egg antigen (schSEA) derived from urine of experimentally confirmed schistosome-infected persons, on normal prostate cells by exploring certain cellular event characteristic of carcinogenesis, including oxidative stress, cell proliferation, and apoptosis [20].

Diagnosis of *Schistosoma spp* infection in asymptomatically and chronically infected persons is a clinically important challenge. Asymptomatically and chronically infected persons pass few eggs in urine and stool as most (eggs) get trapped in tissue; hence, their infection status is often missed by microscopy, which is currently the gold standard [21]. Additionally, as infection levels decline over the years, low level of parasitemia may remain undetected in infected persons who may subsequently develop severe pathology and serve as reservoir of infection [22]. In recent times, alternative technique such as the polymerase chain reaction (PCR) assay has shown potential as an effective method of diagnosis due to its relatively high specificity and sensitivity [23]. In this study, the mitochondrion COX1 gene-based multiplexed PCR technique [24] was seamlessly used for identification and confirmation of the schistosome species infecting the participants. Earlier studies have revealed that some communities nearby the Densu River, including Manheim and Galilea, were endemic for schistosomiasis caused by both *S. haematobium and S. mansoni* [25, 26]. Whereas no participant was detected to have *S. mansoni* infection, only via microscopic evaluation of urine samples, PCR amplification in our study, revealed that 9 participants carried single infections with *S. mansoni*. This is may be attributed to the fact that the infected participants were lightly infected or chronically infected hence passed few or no *S. mansoni* eggs in the urine, thus resulting in lack of detection via microscopy. This also alludes to the high sensitivity of molecular techniques such as PCR than conventional microscopy. However, fewer (21) infected participants were detected by PCR as compared to microscopy, which recorded 30 infected participants. This observation may be due to the likely circulation of genetically hybrid schistosome eggs with similar (“*haematobium*-like or *mansoni*-like”) morphologies as a result of the suspected heterospecific pairing. Contrary to the earlier beliefs that heterospecific (between phylogenetically distant) pairing always resulted in parthenogenetic eggs and offspring, recent studies have experimentally demonstrated that interspecies pairing could generate hybrids (eggs and for that matter offspring) that might remain undetected by conventional PCR [27].

Several *in vitro* studies have identified increased reactive oxygen species (ROS) as initiation agents in cancer [28]. The production of ROS and associated oxidative stress has been implicated in cellular abnormalities such as imbalance of intracellular calcium homeostasis, disruption of membrane lipids, and DNA aberrations [29]. Most importantly, ROS generation in cells elicits a corresponding protecting effect from endogenous cellular antioxidants including GSH and other essential enzymes such as catalase, superoxide dismutase, glutathione peroxidase, and glutathione transferase. Extreme accumulation of oxidants (ROS) may result in depletion and reduction of cellular antioxidant defenses resulting in stress condition that leads to the aforementioned deleterious consequences. Earlier studies [12, 28] have revealed the presence of estradiols in *Schistosoma* eggs and implicated them in pathogenesis of cancer via an oxidative stress route. Enzymes including cytochrome P450 (CYP 1B1) and peroxidases metabolically oxidize these estradiols into highly reactive quinone metabolites [13]. Conjugation of these metabolites with GSH and enzymatic reduction to reform catechol estrogens are the processes by which cells protect themselves from the accumulation of these reactive metabolites. Thus, accumulation of these metabolites results in depletion of GSH and accumulation of GSSG resulting in stress condition. In this study, the observed significant increase in the depletion of prostate cell GSH levels may be attributed to the accumulation of reactive metabolites with increasing concentration of SEA. The probe OPA binds to free cellular GSH to form a highly fluorescent isoindole GSH conjugate [30]. The concentration of GSH in serum-treated cells is relatively high, hence resulting in the observed increase in fluorescence intensity upon treatment with OPA. Conversely, the dose-dependent decrease in fluorescent intensity recorded for the schSEA-treated cells may be attributed to the dose-dependent decrease in GSH levels possibly arising from the metabolic accumulation of reactive metabolites at high concentrations of schSEA which preferentially conjugate to GSH, thus depriving OPA of enough substrate (GSH) for conjugation. This observation is consistent with an earlier study [28] in which HCV 29 (normal urothelial) cells were challenged with *S. haematobium* soluble egg antigens to investigate the mechanism underlying of schistosome-associated bladder cancer.

The oncogenic effects of schistosome infection have been reported in various geographical settings, providing evidence that alterations of certain cell proliferation-associated genes (downregulation of p27, deletion of p16, and mutation in exons 5, 6, 8, and 10 of p53) are responsible for schistosomiasis-associated bladder cancers [14, 31, 32]. Estradiol quinones (the major carcinogen of estrogen) have been implicated as the key genotoxic agent responsible for its ability to form depurinating adducts that have oncogenic consequences [13]. In this study, the cell proliferative effect of schSEA was elucidated by the MTS cell proliferation assay. A dose-dependent increase in absorbance due to increase in cell proliferation was recorded for schSEA-treated cells (up to 12.5 *μ*g/ml concentration) as compared to the serum-starved control. This is attributed to the concentration-dependent increase in the levels of metabolically generated estradiol quinones leading to oncogenic genotoxicities that may be characterized by increased cell proliferation. On the other hand, extreme oxidative stress on cells has implication in programmed cell death (apoptosis) via the intrinsic (mitochondrion-dependent) pathway [33]. Extreme cellular oxidative stress induces the loss of the mitochondrial membrane potential (∆*ψ*) and cytochrome c release from the mitochondria to the cytosol, leading to caspase-9-dependent activation of caspase-3 and finally cell death [34]. Thus, the observed decrease in cell proliferation at the high SEA concentrations (25 *μ*g/ml and 50 *μ*g/ml) may be attributed to the cytotoxic effect of the excessive accumulation of highly reactive estradiol quinone intermediates.

Cancer cells have rewired genetic architecture (upregulated oncogenes and downregulated tumor suppressor genes) that enables them to grow uncontrollably with diminished or inhibited apoptosis [35]. To understand schistosomiasis-associated carcinogenesis, Botelho and colleagues demonstrated that inactivation of p27 and upregulated expression of B-cL2 contributed to cancer hallmarks (mainly stimulated cell proliferation and inhibited apoptosis) in Chinese hamster ovary (CHO) cells challenged with lysates of *S. haematobium* eggs and adult parasites. The p27 (cyclin-dependent kinase inhibitor 1B) is a tumor suppressor protein that regulates cell cycle progression at G1 phase by binding to and inhibiting activation of cyclin D and cyclin-dependent kinase 4 [36]. Its inactivation/downregulation therefore results in unregulated and excessive cell proliferation. On the other hand, B-cL2 (B cell lymphoma 2) is a protein localized to the outer membrane of mitochondria and plays a crucial role in promoting cell survival and antagonizing the actions of proapoptotic proteins (proteins that promote apoptosis).

This phenomenon may explain the observed dose-dependent increase in the percentage of viable cells with corresponding concentration-dependent decrease in the percentage of apoptotic cells in schSEA-treated cells relative to the negative control (non-SEA-treated serum-starved cells).

Apoptosis is a highly regulated and controlled process that is characterized/preceded by distinct cell morphological changes including blebbing, cell shrinkage, global mRNA decay, nuclear fragmentation, and chromatin condensation [37]. These cell morphological changes are triggered by multiple cellular pathways of which two have been well characterized, namely, the intrinsic pathway and the extrinsic pathway [38]. Both pathways are mediated by a group of cysteine proteases known as caspases. Caspases initially exist as zymogens but are activated by proteolytic cleavage of specific fragments [37]. They are classified into initiator caspases (8, 9, and 10) and effector caspases (3, 6, and 7).

Caspase 3 is an effector caspase which upon activation mediates apoptosis by cleaving (inactivating) PARP (poly-ADP ribose polymerase), a protein which plays a key role in cellular assembly and is involved in repair of single-strand DNA breaks. Additionally, activated caspase 3 activates the cytoplasmic endonuclease CAD (caspase-activated DNase) by cleaving off its inhibitory subunit ICAD (inhibitor of CAD) [38]. Activated CAD translocates into the nucleus and cleaves DNA into internucleosomal fragments and induces nuclei condensation/shrinkage, morphological phenotype characteristic of apoptosis. In this study, previously established apoptosis-diminishing effect of schSEA was further investigated by elucidating its effect on nuclei morphology of prostate cells using the DNA/nuclei-intercalating fluorescence dye, Hoechst 33258. Fluorescence microscopic evaluation revealed a dose-dependent decrease in the number of morphologically condensed nuclei in schSEA-treated cells. However, control (untreated) cells displayed numerous morphologically condensed nuclei. This observation suggests that schSEA may induce oncogenic alterations in cells that highly favor cell proliferation and diminish/inhibit activities of key mediators of the apoptosis cascade.

## 5. Conclusion

*Schistosoma spp* soluble egg antigens induced oncogenic phenotypes including oxidative stress, increased proliferation, and diminished apoptosis in cultured normal human prostate cells. This study, for the first time, has provided empirical evidence on the possible role of schistosome infection as an etiological agent in the pathogenesis of prostate cancer. *In vivo* studies including those that involve the use of mouse (BALB/c) model and elucidation of other oncogenic hallmarks such as cell cycle alteration, expression patterns of oncogenes, and tumor suppressor genes would prove informative in establishing this research finding.

## Figures and Tables

**Table 1:** Effect of SEA treatment on proliferation of PNT2 cells.

schSEA (*μ*g/ml)	Cell proliferation values	*p* value
0	0	
3.125	18.83 ± 6.16	0.201
6.25	25.66 ± 3.68	0.091
12.5	32.53 ± 1.48	0.029^∗^
25	−21.84 ± 10.84	0.293
50	−30.53 ± 12.33	0.158

Cell proliferation values are means ± SD. Data are representative of three independent experiments (*n* = 3). Statistical significance by Student's *t*-test: ^∗^*p* ≤ 0.05.

## Data Availability

The datasets used and/or analyzed during the current study are available from the corresponding author on request.
